# Design and Applications of Tumor Microenvironment-Responsive Nanogels as Drug Carriers

**DOI:** 10.3389/fbioe.2021.771851

**Published:** 2021-10-22

**Authors:** Xinjing Du, Yuting Gao, Qi Kang, Jinfeng Xing

**Affiliations:** ^1^ School of Chemical Engineering and Technology, Tianjin University, Tianjin, China; ^2^ Department of Cardiology, Tianjin Medical University General Hospital, Tianjin, China

**Keywords:** nanogels, stimuli-responsive, controlled release, drug delivery, tumor microenvironment

## Abstract

In recent years, the exploration of tumor microenvironment has provided a new approach for tumor treatment. More and more researches are devoted to designing tumor microenvironment-responsive nanogels loaded with therapeutic drugs. Compared with other drug carriers, nanogel has shown great potential in improving the effect of chemotherapy, which is attributed to its stable size, superior hydrophilicity, excellent biocompatibility, and responsiveness to specific environment. This review primarily summarizes the common preparation techniques of nanogels (such as free radical polymerization, covalent cross-linking, and physical self-assembly) and loading ways of drug in nanogels (including physical encapsulation and chemical coupling) as well as the controlled drug release behaviors. Furthermore, the difficulties and prospects of nanogels as drug carriers are also briefly described.

## Introduction

The use of nanogels as carriers for drugs and other molecules has been extensively studied over the past 2 decades (Raemdonck et al., 2009; Li Y. et al., 2015; Mohammadi et al., 2020; Zhao et al., 2021). Generally, nanogels are defined as 3D sub-micron sized hydrophilic polymer networks formed through physical or chemical cross-linking, exhibiting the comprehensive properties of hydrogels and nanoparticles simultaneously. Physical cross-linking mainly includes hydrophobic interactions, electrostatic interactions, hydrogen bonds, and ionic interactions. Chemical method is to produce covalent bonds during the preparation of nanogels (Neamtu et al., 2017). Researchers define the acceptable nanogel size as 10–1,000 nm, while others have reported the optimal size of nanogel for biomedical applications is less than 200 nm (Akiyama et al., 2007). Nanogels are able to absorb a large quantity of water. The cross-linking network of nanogels is considered as a matrix to hold the inner liquid medium, while the absorbed water can be regarded as a filter medium for the diffusion of cargoes. Nanogels with negative Zeta potential are beneficial to avoid phagocytosis of the immune system and can resist the adsorption of negatively charged proteins (Xiao et al., 2011; Cuggino et al., 2019). Moreover, the swelling and shrinking behavior of nanogels is considered as an indispensable feature, which promotes the diffusion of loaded drug (Clegg et al., 2020).

With the exploration of controlled drug delivery, many kinds of drug carriers have attracted wide attention, such as liposomes, polymer vesicles, micelles, and microemulsions due to their good performances in prolonging blood circulation and improving therapeutic efficiency (Zhu et al., 2017; Iqbal et al., 2020; Large et al., 2021). As an emerging drug carrier, nanogel shows unique and promising prospects in biomedical field because of its good biocompatibility, high drug loading capacity, stimuli-responsiveness, low toxicity, and biodegradability (Pinelli et al., 2020; Shah et al., 2020). In general, an ideal nanogel drug delivery system (NG-DDS) should meet the requirements of the entire drug delivery process. Firstly, nanogels are required to effectively load therapeutic drugs and protect them from phagocytosis, elimination, and burst release. Secondly, nanogels should target to the diseased tissues to reduce damage to normal cells. Afterwards, under corresponding stimulus (temperature, pH, magnetic field, light, redox potential, enzymes, etc.), the structures of nanogels are triggered to collapse, swell, or contract to achieve controlled drug release. Finally, the remaining nanogels should be degradable and eliminated from the body with circulation (Xu et al., 2013; Ahmed et al., 2020). Therefore, the design strategies of nanogels as drug carriers include high drug loading content, good biocompatibility, long circulation time, specific ligands recognized by targeted cells, and stimulus-sensitive degradation characteristics.

**SCHEME 1:**
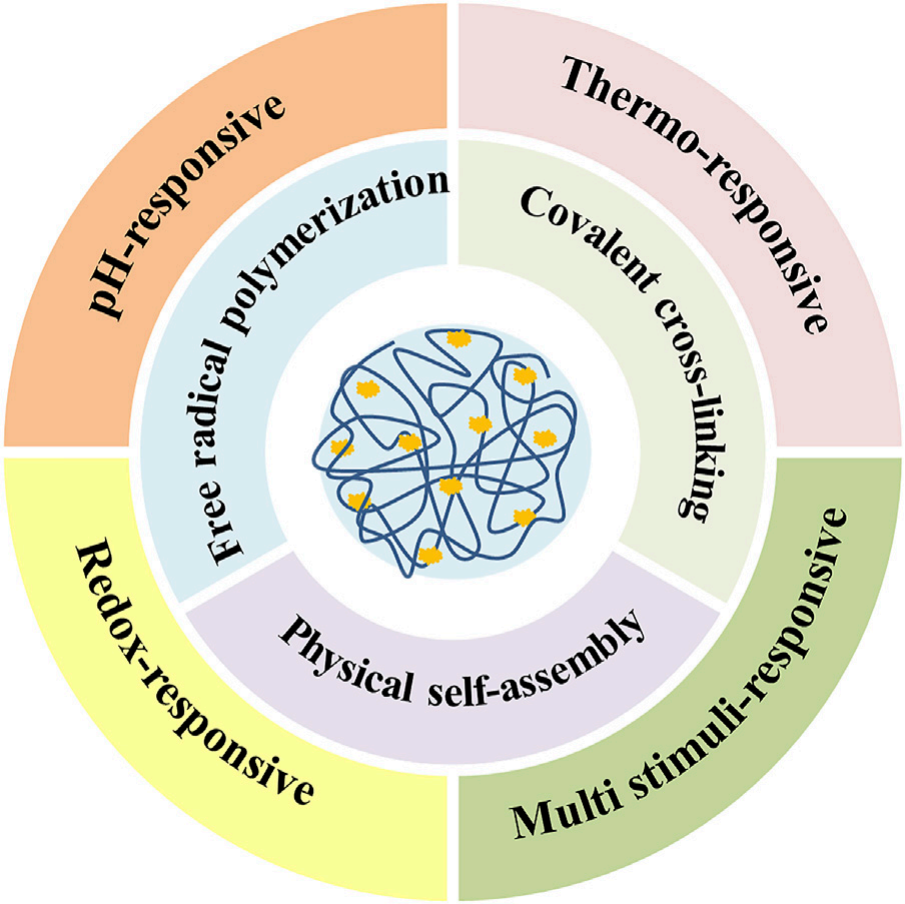
General preparation methods and responsive-release types of drug-loaded nanogels.

In recent years, researchers have developed various nanogels with abundant performances. Most nanogels are designed to load therapeutic drugs such as doxorubicin (DOX), curcumin (CUR), methotrexate (MTX), and cisplatin (CDDP), and the others are utilized to deliver nucleic acids, proteins, and genes (Li et al., 2017; Luckanagul et al., 2018a; Lakkakula et al., 2021). So far, the instability of carriers and the presence of various biological barriers are still the main challenges to drug delivery efficiency (Oh et al., 2008; Sharma et al., 2016; Mauri et al., 2018; Hajebi et al., 2019b; Dreiss, 2020). In order to further explore NG-DDS comprehensively and systematically, this review primarily summarizes the synthetic strategies of nanogels (chemical crosslinking and physical crosslinking), methods of drug loading (physical encapsulation and chemical coupling) as well as stimuli-responsive drug release behaviors (pH, temperature, redox; single, dual, and multi). In addition, according to the development of NG-DDS, major challenges and future prospects of NG-DDS are also depicted (Scheme 1).

## General Synthetic Methods

The preparation of nanogels is divided into chemical methods and physical approaches. Chemical methods involve the formation of covalent bonds and the most common technology is the heterogeneous free radical polymerization (Li Z. et al., 2019; Mackiewicz et al., 2019; Gurnani and Perrier, 2020). Compared with traditional free radical polymerization, controlled living polymerization could synthesize polymers with specific structures and properties (Gurnani and Perrier, 2020). In order to control the molecular weight, particle size, and particular three-dimensional structure of the polymer, atom transfer radical polymerization (ATRP), reversible addition-fragmentation chain transfer (RAFT), and nitroxide radical polymerization (NMRP) have been widely developed. Besides, the introduction of special cross-linkers into the polymerization system also tends to construct covalently cross-linked nanogels. The decomposition of cross-linkers is beneficial to the degradation of nanogels and controlled drug release in the later stage (Pei et al., 2018). Physical cross-linking means the self-assembly of polymers through weaker interactions, including hydrophobic interactions, supramolecular host-guest assembly, electrostatic interactions, and hydrogen bonds (Wei et al., 2013; Ding et al., 2019b). Comparing these two procedures, the chemical cross-linking shows more permanent and stable connections for the polymer network, whereas physically cross-linked structure is more likely to be destroyed. It is notable that the different cross-linked structures also influence the subsequent ways of drug release. A list of cross-linking mechanisms and corresponding release behaviors of drug-loaded nanogels is shown in Table 1.

**TABLE 1:** The preparation mechanisms and relevant drug release behaviors of NG-DDS.

Network structure	Cross-linking mechanism	The nanogel size	The loaded molecule	Stimuli-responsive drug release	References
Chemical cross-linking	Traditional free radical polymerization	90–230 nm	Doxorubicin	pH/thermo	Wang et al. (2015)
	Controllable/living free radical polymerization	82–153 nm	Coumarin 102	UV light	Xin et al. (2020)
	Disulfide, imine cross-linking	250 nm	Doxorubicin	pH/redox	Pei et al. (2018)
	Click reaction	236 nm	Labeled insulin	Glucose/H_2_O_2_	Li et al. (2019a)
Physical cross-linking	Hydrophilic and hydrophobic interactions	55.7–259.2 nm	Nile Red	pH	Wang et al. (2018)
	Supramolecular host-guest assembly	108.1–121.4 nm	Doxorubicin and indocyanine green	NIR light	Zan et al. (2015)
	Hydrophobic/electrostatic interactions	140–230 nm	Curcumin	pH/oxidation	Dong et al. (2020)
	Hydrogen bonds	230 nm	DAPI, fluorescein, Nile red	Thermo	Zavgorodnya et al. (2017)
Both	Disulfide cross-linking/hydrophobic interactions	66.95 ± 1.93 nm	Purpurin 18 and 10-hydroxycamplothecin	Redox	Ma et al. (2021)
	Free radical polymerization/hydrogen bonds	18 nm	Curcumin	Thermo/NIR light	Wang et al. (2014)
	Free radical polymerization/disulfide cross-linking/ electrostatic interactions	70 nm	Doxorubicin	pH/thermo/photo	Chen et al. (2017)

### Free Radical Polymerization

**FIGURE 1:**
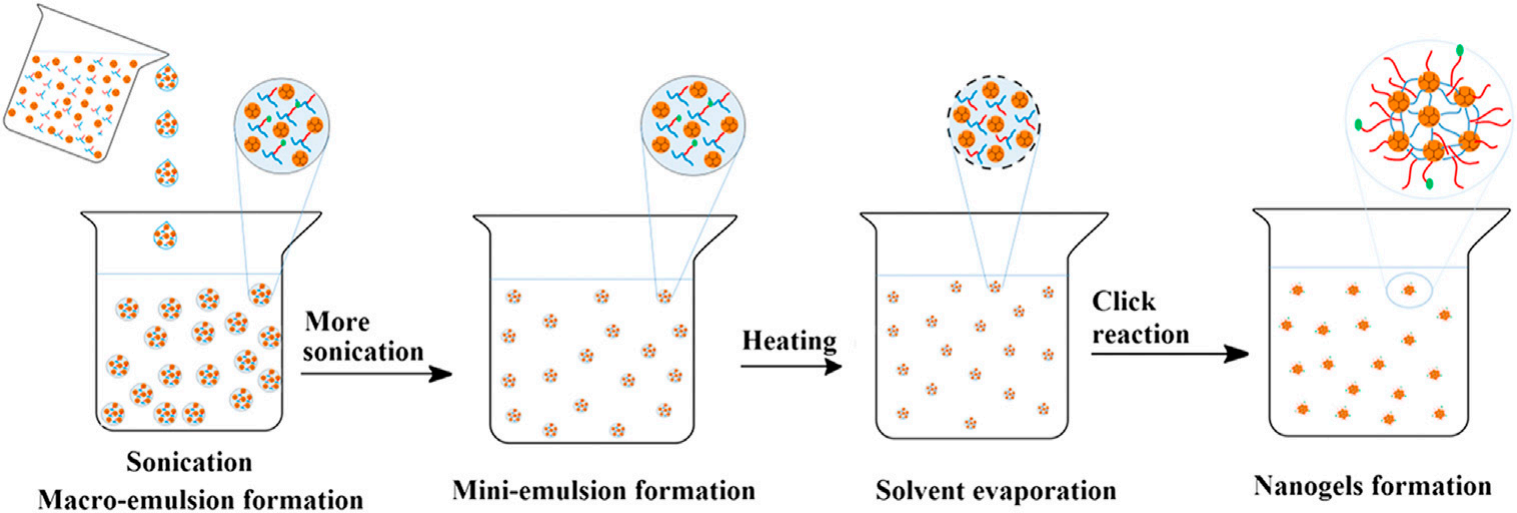
Schematic illustration of the mini-emulsion technique (Abandansari et al., 2014). Copyright (2014) Elsevier.

Most nanogels are prepared by free radical polymerization, which have the advantages of fast reaction speed, high molecular weight of the products, and the increasing of conversion rate with the extension of reaction time (Noordergraaf et al., 2018; Gao et al., 2020). The structures and properties can be adjusted by changing monomer, crosslinking agent, initiator, reaction medium, reaction time, and reaction temperature to achieve optimal drug delivery effect (Ahmed, 2015). Precipitation polymerization, (micro) emulsion polymerization, and dispersion polymerization are common polymerization techniques. Utilizing N-isopropylacrylamide as the monomer, acrylic dendritic polyglycerol as the crosslinking agent, sodium lauryl sulfate as the stabilizer, temperature-sensitive nanogels loading coumarin six were prepared by precipitation polymerization. The results showed that the drug release was thermo-dependent with a remarkable increase above the volume phase transition temperature of 32–37°C (Sahle et al., 2017). Sengel prepared a drug carrier with N-(2-mercaptoethyl)acrylamide as a monomer and ethylene glycol dimethacrylate as a cross-linker through dispersion polymerization (Sengel and Sahiner, 2019). Emulsion polymerization means monomers are dispersed in water with the help of emulsifiers and mechanical stirring, and micelles are formed above the critical micelle concentration, where polymer chains keep sustained growth. On this basis, micro (mini) emulsion polymerization and reverse (micro) emulsion polymerization have been developed (Lovell and Schork, 2020; Pereira et al., 2020). The pH-sensitive H40-based nanogels with new structures were synthesized through mini-emulsion polymerization and click reaction (Abandansari et al., 2014). Firstly, the synthesized reactants were added dropwise to the aqueous phase with sonication to obtain the milky macroemulsion. Then the formed macroemulsion was constantly ultrasonicated under ice cooling to form a stable milky miniemulsion. Subsequently, the miniemulsion was heated and catalysts were added to induce the azide-alkyne click reaction and the pure nanogels were collected through dialysis (Figure 1).

With the development of free radical polymerization and controlled drug delivery systems, controllable/living free radical polymerization has attracted much attention (Matyjaszewski and Xia, 2001; Kim et al., 2016; Lou et al., 2017). These prepared polymers have the characteristics of narrow molecular weight distribution and uniform particle size distribution, which facilitate the construction of homogeneous polymer networks and drug loading. ATRP relies on external catalysts (usually transition metal complexs) to reversibly deactivate the free radicals to a dormant state (Matyjaszewski and Xia, 2001). Most of the biodegradable, well-defined and water-dispersed nanogels prepared by ATRP technology occur in reversed micro (mini) emulsions (Siegwart et al., 2009; Averick et al., 2011). The disulfide crosslinked biodegradable nanogels were designed and synthesized employing inverse miniemulsion ATRP (Oh et al., 2007). The releases of encapsulated molecules such as the fluorescent dye rhodamine and the anticancer drug Dox were triggered by the biodegradation of nanogels, demonstrating that these nanogels can be developed as targeted drug delivery carriers for biomedical applications. Compared with ATRP, RAFT with simpler procedures usually uses chain transfer agent (thiocarbonyl compound) rather than poisonous catalysts (Xin et al., 2020). The poly (methyl methacrylate) hair nanoparticles with core-shell structures were prepared through RAFT method. The carriers had high passive drug loading capacity for DOX, exhibiting fast and adjustable drug release behavior at intracellular pH (Qu et al., 2017). It is noticeable that although there are lots of merits of controlled living free radical polymerization, these preparations are rarely studied *in vivo*, and there is little information about biodistribution, clearance and long-term tolerance.

At present, photo-initiated polymerization has become an effective preparation method due to the short reaction time, mild reaction conditions, and controllable time and space (Fu et al., 2015). Messager reported a synthetic method for hyaluronic acid-based nanogels with controllable structures (Messager et al., 2013). Under UV light irradiation, the methacryloyl hyaluronic acid precursor started crosslinking in the droplets of water-in-oil emulsion, which obtained nanoparticles with homogeneous sizes. However, UV radiation may cause potential cell damage, and UV light tends to be scattered by large monomer droplets. Therefore, visible light-induced photopolymerization has been developed because they have longer wavelengths, less scattered by larger objects and better penetration (Matsui et al., 2017; Le Quemener et al., 2018). For example, Bakó prepared nanogels using methacrylic acid poly-γ-glutamic nanoparticles loading antibiotic drug ampicillin by visible (blue) light-initiated photopolymerization, and the release kinetics showed the controlled and efficient release behaviors (Bakó et al., 2016). Notably, our lab has also developed some strategies on the preparation of nanogels through laser photopolymerization, which is related with the investigation of the initiating system, polymerization mechanism and biomedical applications (Li J. et al., 2021; Liu et al., 2021; Peng et al., 2021; Wang et al., 2021). Specifically, Wang chose biocompatible polyethylene glycol diacrylate (PEGDA) as a monomer and ultrasmall nanogels with around 30 nm in size were prepared successfully through surfactant-free photopolymerization at 532 nm. Subsequently, Li in our lab introduced the third component DPI into the EY/TEOA initiating system, which significantly increased the polymerization rate and conversion ratio, and multifunctional PEGDA hydrogels through a beam expansion device were investigated. Liu’s work concentrated on rapid preparation of nanogels through laser beam expansion under low monomer concentration. And the role of triethanolamine in the effect on the cross-linking degree of PEGDA nanogels was investigated by Peng, indicating that triethanolamine could adjust the double-bond conversion.

### Covalent Cross-Linking

The networks of the nanogels can be obtained not only by the polymerization of C = C bonds, but also by coupling between other groups. Compounds containing disulfide bonds are one of the most commonly used cross-linking agents, because the disulfide bonds are easily broke by high concentrations of glutathione in tumor tissues and cells to achieve redox-responsive drug release. For instance, Tian and his coworkers prepared self-assembled hyaluronic acid and polyethylene glycol diglycidyl ether nanogels, then added with cystamine for the second cross-linking and loaded with DOX into the dense networks (Tian et al., 2019). Double cross-linking structures increased the tightness and decreased the burst release of drugs. Besides, the nanogels containing imine bonds formed by aldamine condensation are extensively utilized to deliver drugs due to their good biological activity and acid-sensitivity (Su et al., 2016; Zhao et al., 2017). The modified alginate was coupled with cystamine *via* disulfide bonds and coupled with DOX *via* imine bonds, achieving folate receptor-mediated targeting and pH/reduction dual-responsive drug release (Pei et al., 2018). The cleavage of unstable disulfide bonds and imine triggered the collapse of nanogels, promoting the DOX release and accumulation in the nucleus. In particular, these nanogels exhibited strong fluorescence in acidic media such as the microenvironment of tumor cells, and could be used for real-time, non-invasive positioning and tracking of cancer cells.

**FIGURE 2:**
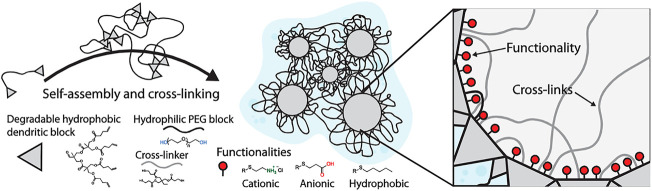
Purposed structure of dendritic nanogels (DNGs) (Zhang et al., 2019). Copyright (2019) John Wiley and Sons.

In recent years, click chemistry has become a promising strategy for designing nanogels due to its high reactivity, good selectivity, and mild reaction conditions. The thiol-ene click reaction is common in the addition of thiol to the double bonds under photo-initiation, which is efficient, high-yield, and can tolerate different functional groups (Tasdelen et al., 2016; Le et al., 2018; Phan et al., 2020; Mondal et al., 2021). The PEG and polycyclic phenylborate nanogels loaded with insulin and glucose oxidase (Gox) were developed through a one-pot thiol-ene click chemistry method by using tetrathiol compound QT as a coupling agent (Li C. et al., 2019). Since the H_2_O_2_ produced during the oxidation of glucose could cause cytotoxicity, the *in-situ* consumption of H_2_O_2_ effectively alleviated the side effects of Gox. Compared with nanogels without Gox, nanogels loaded with Gox presented better glucose-responsive abilities and stronger insulin release. These novel glucose/H_2_O_2_ dual-sensitive nanogels for insulin delivery will be a promising candidate material in the treatment of diabetes in the future. Zhang reported the self-assembly of modified PEG with hydrophobic segments through hydrophilic and hydrophobic interactions and cross-linked with thiols as shown in Figure 2 (Zhang et al., 2019). The loading capacity of nanogels for DOX, gemcitabine, and methotrexate was investigated, showing that all three drugs could be successfully encapsulated inside the nanogels, and delivered to the 3D pancreatic spheroid tumor model. In summary, many researches have demonstrated that click nanogels with good biocompatibility are very suitable for targeted intracellular drug delivery. However, the current research is still in the preliminary stage. In-depth exploration and reasonable design are necessary to realize true clinical applications.

### Physical Self-Assembly

**FIGURE 3:**
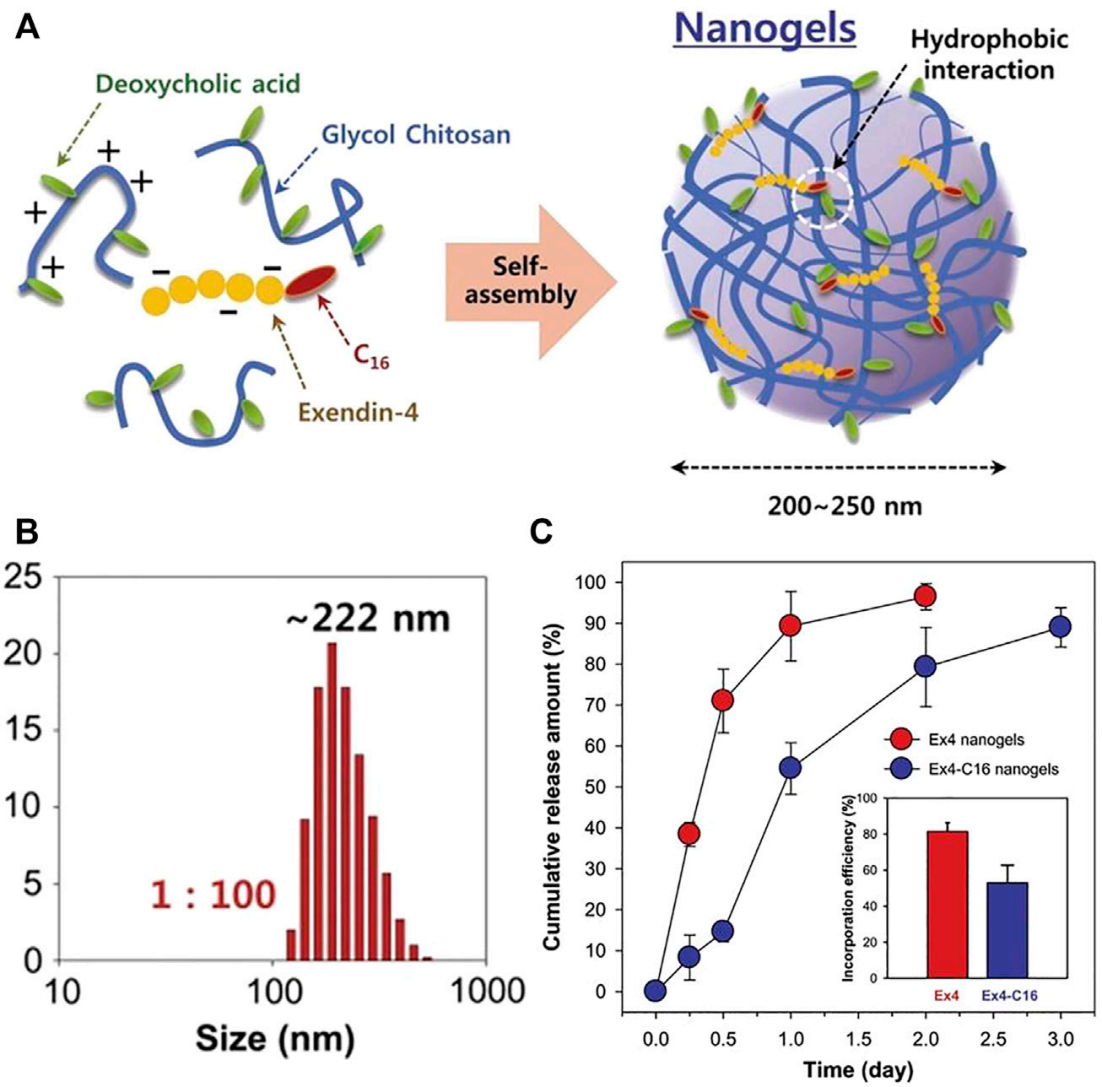
Preparation illustration of nanogels and drug release behaviors. **(A)** An illustration of self-assembled nanogels; **(B)** The nanogel particle size distribution with ratio 1:100; **(C)** Release characteristics of Ex4 and Ex4-C16 from nanogels (Lee et al., 2012). Copyright (2012) Elsevier.

Compared with chemical cross-linked nanogels, nanogels prepared by physical cross-linking show new functions and have the potential to process, recycle and self-repair due to the nature of dynamic and reversible non-covalent interactions. Usual preparation techniques are to couple hydrophobic segments to hydrophilic polymers, forming amphiphilic macromolecules, and then nanogels will be obtained *via* self-assembly in aqueous solutions (Chattopadhyay et al., 2016; Thelu et al., 2018; Wang et al., 2018; Wu et al., 2018; Wang et al., 2020). The self-assembled nanogels of ethylene glycol chitosan modified with deoxycholic acid were synthesized, and the diabetic drug palmitoylated exendin-4 (Ex4-C16) was adsorbed on the deoxycholic acid of the chitosan nanogels by hydrophobic interactions (Lee et al., 2012). This kind of drugs could combine with serum albumin to avoid the filtering effect of kidneys and prolong the half-life in the blood. Compared with natural Ex4, palmitoylated Ex4 nanogels had lower penetration rate, but the drug release was slower with longer hypoglycemic effects. These nanogels produced hypoglycemia for 2 days in diabetic mice at a relatively low dose (100 nmol/kg), indicating that the nanogels had long-term hypoglycemic abilities as displayed in Figure 3. In a word, these drug carriers synthesized by hydrophobic interaction are beneficial to the adsorption of hydrophobic drugs, and the interactions between the drugs and carriers can also delay the drug release.

**FIGURE 4:**
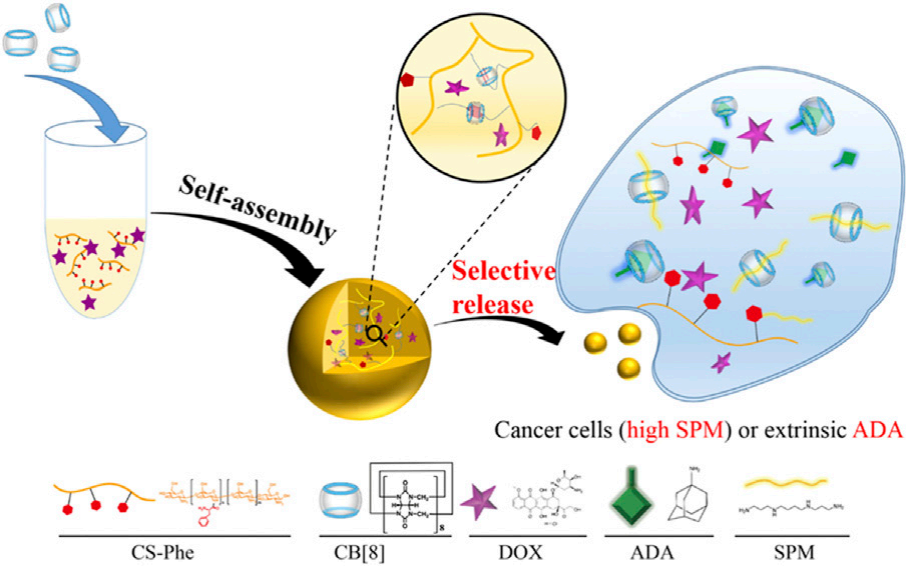
Scheme showing the preparation of host-guest interaction-initiated supramolecular CNGs and their stimuli-responsive payload release in cancer cells (Ding et al., 2019b). Copyright (2019) American Chemical Society.

Supramolecular chemistry refers to the chemistry of molecular aggregates based on non-covalent bond interactions between molecules, which primarily studies weak interactions and the assembly, structure, and performance of molecular aggregates (Zan et al., 2015; Qin et al., 2020). Compared with traditional covalently cross-linked nanogels, supramolecular nanogels are easier to adjust their chemical and physical properties by changing the ratio of different components or introducing different stimulus-responsive groups. Common supramolecular host molecules include cyclodextrin, calixarene, crown ether, and hyperbranched polymer (Webber et al., 2016). According to Ding, the phenylalanine grafted chitosan and DOX were wrapped in the cavity of cucurbit(8)urea [CB(8)] to prepare chitosan nanogels with high drug loading efficiency, good biocompatibility, and selective cytotoxicity against therapeutic targets (Ding et al., 2019a). The CB cavity here functioned as a cross-linking agent, and the self-assembly of macromolecules was realized by wrapping phenylalanine in the cavity. Over-expressed spermine or exogenous stimulus amantadine in tumor cells would replace phenylalanine, because the binding affinity of phenylalanine to CB (8) was much weaker than that of amantadine or spermine to CB (8), leading to decrosslinking of nanogels. This kind of drug carriers could respond to specific endogenous or exogenous stimuli to achieve controlled drug release (Figure 4).

Particularly, ionically cross-linked nanogels are provided with better stability than self-assembled nanogels with micellar structures (Perez-Alvarez et al., 2016; Dong et al., 2020). Ionic polysaccharides are commonly used natural macromolecules for the synthesis of nanogels, including chitosan, hyaluronic acid, alginate, etc. (Liang et al., 2015). Nanogels loaded with antitumor drug methotrexate (MTX) were synthesized through ion gel methods using chitosan and sodium tripolyphosphate (TPP) with the small average particle size of 53.34 ± 7.23 nm, which helped nanogels extend blood circulation time and crossed the blood-brain barriers. After coating the nanogels with polysorbate 80, the MTX-loaded nanogels showed sustained drug release behaviors (about 65–70% within 48 h). These surface-modified nanogels might be candidates for drug delivery to the central nervous system (Azadi et al., 2012). Besides, alginate could be mixed with cationic poly [(2-dimethylamino) ethyl methacrylate] in water through electrostatic attraction, forming nanogels with size of 150 nm. When loading DOX, the nanogels exhibited acid-accelerated release behaviors as a result of the protonation of DOX and nanogels in an acidic medium, facilitating drug release due to electrostatic repulsion. This preparation method was simple in operation, and had low cost, no organic solvent or additives, good biocompatibility, and controllable drug release, which is beneficial to the drug delivery (Cai et al., 2012).

### Microfluidic Technology

Unlike traditional methods, microfluidic technology has precise fluid control and rapid micro-scale mixing, which has aroused widespread interest in the preparation and engineering of nano-drug delivery materials. Compared with the traditional batch method, the drug-loaded nanomaterials prepared by the microfluidics have better monodispersity and their microstructures could be controlled by changing the flow rate and time (Amrani and Tabrizian, 2018; Nie et al., 2019; Zhang et al., 2020). To solve the issues of nanogels as carriers with large size, poor cell uptake rate, and endoplasmic embedding, Huang reported the preparation of HA-based nanogels with particle size of 80–160 nm *via* microfluidic technology and tetrazole-olefin light click cross-linking (Huang et al., 2019). The microfluidic chip was made of soda lime glass and nano-droplets formed at the intersection of the three water inlets. Within a certain range, the particle size decreased as the flow rate increasing and the concentration of HA reducing. These carriers could load various therapeutic proteins, such as cytochrome C, herceptin, and BSA with reduction-sensitivity because of the broken of disulfide bonds in the presence of glutathione.

Besides, microfluidic synthesized nanogels could show superior performance in the encapsulation and release of loaded cargoes (Rhee et al., 2011; Feng et al., 2015). For example, the alginate-based and growth factor-loaded nanogels were prepared through ionic gelation using the microfluidic technology (Mahmoudi et al., 2020). An alginate solution mixed TGF-β3 as core flow was injected into the microchip, while CaCl_2_ as the sheath flow was injected into the side channels’ inlets of microchip, resulting in the interaction and rapid ionical cross-linking between alginate polymer chains and Ca^2+^ ions. According to the dynamic light scattering analysis, the microfluidic synthesized nanogels had a smaller diameter and a better polydispersity index (43 ± 4 nm, PDI ≤ 0.2) than those of the bulk prepared nanogels (137 ± 22 nm, PDI ≥ 0.5). And the sizes of nanogels would increase with the enhancement of the flow rate ratio, showing a good correlation. Based on the small diameters and compact characteristics of nanogels, the high encapsulation efficiency and slow drug release could be achieved, while the large sizes of the bulk synthesized nanogels caused the low encapsulation efficiency and burst release. Therefore, the nanogels prepared through the microfluidic approach have shown good potential in cargo loading and sustained release.

## Loading Ways of Drug in Nanogels

Loading content refers to the amount of drug loaded per unit weight or unit volume of nanogel, and the amount of drug that can be released is the effective drug loading. An efficient nanocarrier system is required to have high drug loading content, because the drug loading capacity directly affects the clinical application (Sun et al., 2017). The loading methods of carriers include non-covalent and covalent means, namely physical packaging and chemical conjugation. A list of drug-loading ways, the encapsulation techniques and corresponding release behaviors of drug-loaded nanogels is shown in Table 2.

**TABLE 2:** The drug-loading ways and relevant drug release behaviors of NG-DDS.

The drug-loading way	The loaded drug	The encapsulation technique	The encapsulation efficiency (%)	Stimuli-responsive drug release	References
Physical encapsulation	Chlorin e6	The dialysis method	61.9 ± 0.15	Hyaluronidase	Yoon et al. (2012)
	Curcumin	The sonication method	With 114% loading of curcumin over the solution	Temperature	Luckanagul et al. (2018b)
	Doxorubicin	Hydrogen-bonded complexes	82 ± 4	Glutathione	Senthilkumar et al. (2019)
Chemical coupling	Chlorin e6	Amidation reaction	96.23 ± 4.8	—	Lee et al. (2011)
	Doxorubicin	Schiff base formation	32.66	pH	Su et al. (2018)
	Camptothecin	Esterification reaction	95.4 ± 0.9 (grafting rate)	pH/redox	Qu et al. (2019)

### Physical Encapsulation

Most drugs are loaded in nanogels by non-covalent interactions, including hydrophilic and hydrophobic interactions, hydrogen bonding interactions, and electrostatic interactions, because this method is simple and effective, and does not change the activity of drug molecules (Feng et al., 2010). Among them, the hydrophobic interaction is widely applied, because many therapeutic drugs are hydrophobic that can be adsorbed to the hydrophobic center (mainly in the core) of the nanogels. For instance, Yoon prepared hyaluronic acid nanogels coupled with a hydrophobic segment of 5β-cholanic acid through self-assembly and loaded with chlorin e6 (Ce6) as a hydrophobic photosensitizer with great drug loading content of 80% (Yoon et al., 2012). At the same time, hydrogen bond-based drug loading is also promising due to directionality, selectivity, and relatively strong interaction, providing drug carriers with stronger drug loading capacity and high physical stability (Li et al., 2018). A novel preparation method of three-hydrogen bonds drug conjugated nanogels was developed (Senthilkumar et al., 2019). The hydrogen bond connection achieved a high drug loading ratio (82 ± 4%), while this number was merely 59 ± 3% when drug was loaded through hydrophobic interaction. Importantly, the uniform connection of the hydrogen-bonded conjugates in the network assisted the drug to stay in the gel matrix for long time during the circulation. These nanogels had strong stability, good biocompatibility and achieved sustainable drug release for several days. This new strategy of three-point hydrogen bonds drug coupling could be extended to other carrier systems and various amphiphilic conjugated polymers.

### Chemical Coupling

**FIGURE 5:**
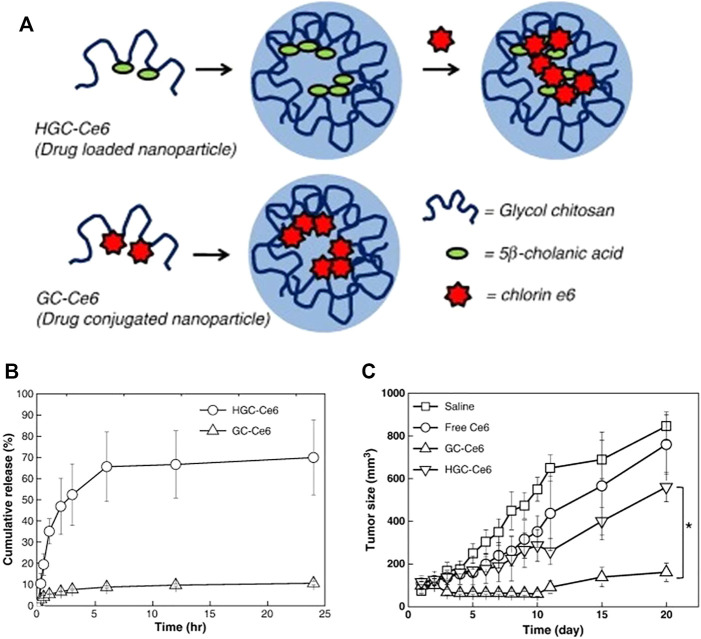
Preparation of HGC-Ce6 and GC-Ce6 and release profiles and *in vivo* therapeutic efficacy. **(A)** Schematic illustration of HGC-Ce6 (drug-loaded nanoparticle) and GC-Ce6 (drug-conjugated nanoparticle); **(B)** *In vitro* release profiles of HGC-Ce6 and GC-Ce6; **(C)** Tumor growth data after photodynamic therapy with free Ce6, HGC-Ce6, or GC-Ce6 (5 mg/kg of Ce6) in HT-29 tumor-bearing mice (Lee et al., 2011). Copyright (2011) Elsevier.

Physical encapsulation usually leads to inevitable burst release, which reduces the therapeutic effect of drugs (Seidi et al., 2018). To overcome this shortcoming and improve the blood circulation time and accumulation of drugs, covalent coupling has been developed. Lee studied the difference between physical loading and chemical coupling of photosensitizers of tumor-targeted glycol chitosan nanogels as shown in Figure 5 (Lee et al., 2011). On the one hand, the hydrophobic photosensitizer Ce6 was physically loaded into hydrophobically modified carriers. On the other hand, Ce6 was chemically coupled with ethylene glycol chitosan and then nanocarriers were synthesized through self-assembly in aqueous solution. Both nanogels had similar particle sizes and singlet oxygen generation efficiency, but the physical loading exhibited sudden drug release under buffer conditions, where 65% of drugs were released rapidly within 6.5 h, while the chemical coupling presented longer circulation time and more effective tumor accumulation. When two drug carriers and free Ce6 were injected into tumor-bearing mice, only the chemically coupled carriers showed good phototoxicity, and the tumor volume in mice exhibited sharper reduction than others. The high-efficiency therapeutic effect was probably related to the chemical bond between the drugs and nanogels, indicating that this kind of NG-DDS was an effective and promising Ce6 delivery system. In addition, the DOX with active amino groups in the molecules is often coupled to nanogels through Schiff base bond. The pH-sensitive drug-loaded nanogels were obtained through coupling hydroformylated dextran nanogels and DOX (Su et al., 2018). At pH 2.0, 5.0, and 7.4, the drug release amount within 72 h were about 66, 28, and 9%, while the physically loaded nanogels did not demonstrate acid-accelerated release behaviors. In general, although chemical methods slightly solve the problem of drug burst release, some chemical reactions may also affect the activity and efficacy of drugs. It can be observed that these two drug loading methods have respective advantages and disadvantages.

## Stimuli-Responsive Drug Release Behaviors

**FIGURE 6:**
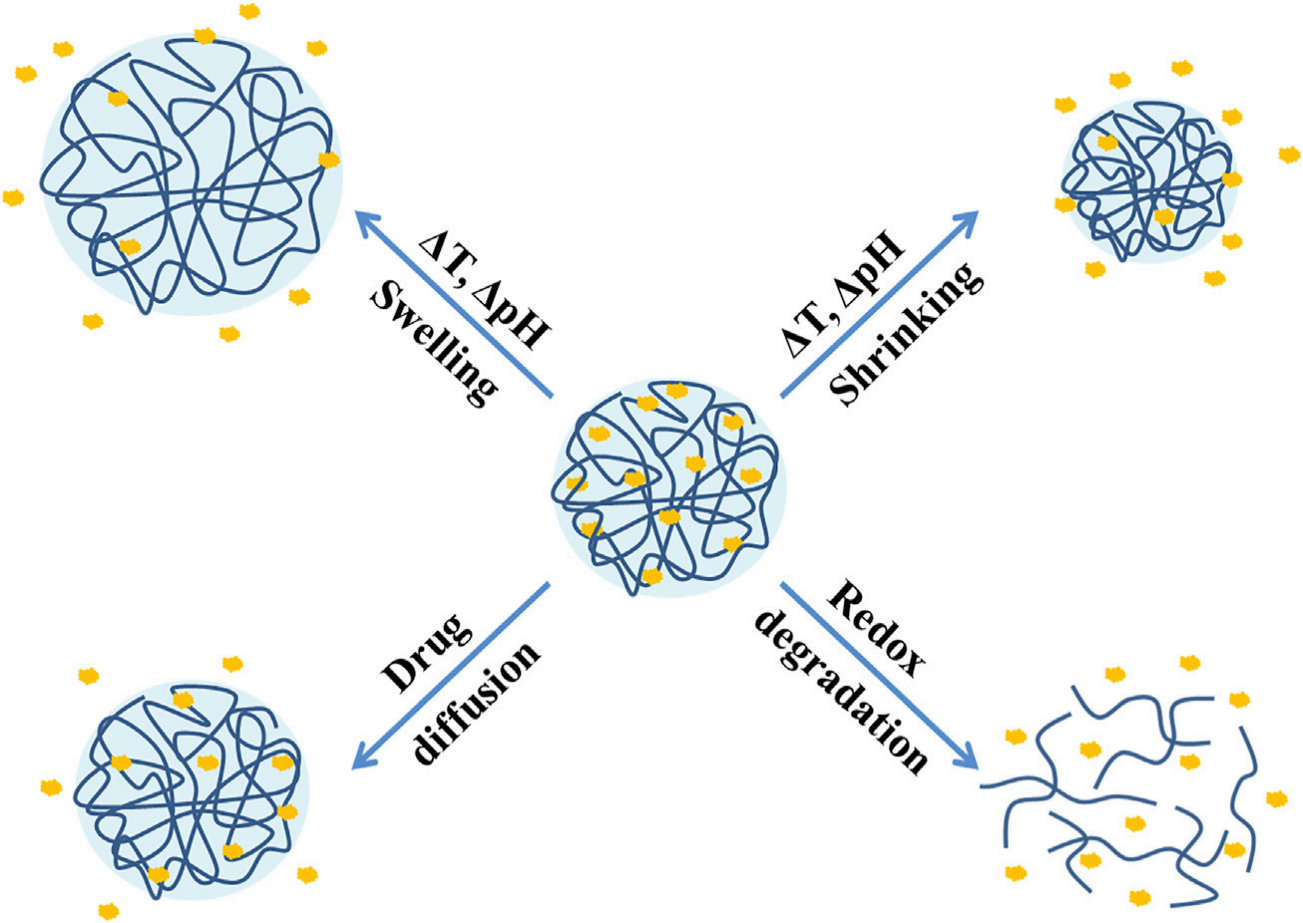
Stimuli-responsive drug release mechanisms of nanogels.

As mentioned in the first section, an ideal NG-DDS needs to achieve effective drug release at the target site, and the most common release mechanism is drug diffusion (Clegg et al., 2020). In fact, the developments in recent years have shown that regardless of physical embedding or covalent coupling, the current release mechanism tends to be stimuli-responsive release (such as pH, temperature, redox, light, enzymes, etc.), which mainly divides into drug diffusion and swelling, shrinkage, and degradation of nanogels as described in Figure 6. Several stimuli-responsive release systems will be introduced in the following.

### pH-Responsive Nanogels

Designing pH-responsive NG-DDS is one of the most commonly used strategies in tumor treatment, because the weakly acidic pH (∼6.5) of tumor cells caused by the excessive lactic acid produced by hypoxia is slightly lower than that (∼7.4) of normal tissues. Furthermore, the acidic lysosomes in cells (pH 5.0–5.5) are also beneficial to the acid-responsive drug release (Li Z. et al., 2021). To obtain acid-sensitive drug carriers, the modification of pH-sensitive molecules and introduction of pH-sensitive bonds (such as acetal bonds, ketal bonds, and imine bonds) into the nanogels are widely used.

Manchun used glyoxal as a crosslinking agent to synthesize dextrin-based nanogels with acid-sensitive bonds (acetal bond) through emulsion polymerization, loaded with DOX as a model drug, and the drug release behaviors of nanogels with different crosslinking agent content in different pH were investigated (Manchun et al., 2014). When the molar ratio of dextrin and glyoxal was 20:1, the cumulative release amount was about 40, 94, and 100% within 72 h at pH 7.4, 6.8, and 5.0, which presented obvious acid-accelerated release behaviors. Under the same pH conditions, as the increase of molar ratio of dextrin and glyoxal, the release amount was accordingly enhanced, because the content of the crosslinking agent was relevant to the crosslinking density of the nanogels. Afterwards, the author replaced glyoxal with formaldehyde as a crosslinking agent, and exhibited similar acid-responsive drug release (Manchun et al., 2015). Moreover, 2,2-dimethylacryloyloxy-1-ethoxypropane (DMAEP) containing ketal bonds was also employed as a pH-labile crosslinking agent. Acid-responsive DOX-loaded nanogels were formed through free radical copolymerization, using acylated HA as a monomer and DMAEP as a cross-linker, which accelerated the DOX release under acidic conditions (Luan et al., 2017). Borate also has unique acid sensitivity, making borate bonds attractive as driving force for integrated assembly. For example, Zhu reported the self-assembly of dextran and phenylboronic acid-modified cholesterol to synthesize DOX-loaded lysosome-acid targeting drug carriers. At the cellular level, it was clearly demonstrated that lysosomes had a strong influence on the uptake efficiency of nuclear drugs, indicating that lysosomal acidity was the main factor affecting drug efficacy (Zhu et al., 2015).

Currently, nanogels with carboxyl or/and amino groups in the molecular structure are commonly used as pH-responsive drug delivery vehicles. The pH and thermo sensitive nanogels with DOX loading composed of poly (N-isopropylacrylamide-co-acrylicacid) core, a polydopamine layer and an outer folic acid layer were designed and developed (Pu et al., 2021). With the decrease of pH from 7.4 to 5.5, the cumulative DOX release amount improved from 17.6 to 56.5%. The enhancement of drug release at pH 5.5 was attributed to the reduction of carboxyl ionization, which induced electrostatic interaction between carboxyl groups as well as the shrinkage of nangels. Moreover, poly (N,N-dimethylaminoethyl methacrylate) (PDMAEMA) is also a pH-sensitive cationic polymer because of protonation of the amino groups when the pH is lower than pKa of PDMAEMA (approximately 7.5) (Brannigan and Khutoryanskiy, 2017; Li Z. et al., 2021). Injectable PDMAEMA nanogels were developed through facile precipitation polymerization with *in situ* thermogelling behaviors and pH-triggered switching activity (Maiti et al., 2018). According to the release profile, the DOX release was acidic dependent from merely 43% at physiological pH to around 80% at pH 5.8 after 96 h of incubation. On the one hand, the protonation of the amino group resulted in increased electrostatic repulsion, causing the swelling of PDMAEMA nanogels. On the other hand, it might originate from enhanced electrostatic repulsion between quaternized PDMAEMA and positively charged DOX drug molecules, facilitating the DOX release.

### Temperature-Sensitive Nanogels

Another feature of tumors and inflammatory areas is that the temperature (40–45°C) is slightly higher than that (37°C) of body fluids, which makes it valuable to design thermo-sensitive nanogels (Seo et al., 2012). Changes in temperature can reverse the segment-segment interaction or the segment-solvent molecule interaction, resulting in swelling or shrinkage of nanogels to achieve responsive drug release with corresponding temperature of the lower and higher critical solution temperature (LCST/UCST) (Yu et al., 2021).

Poly (N-isopropylacrylamide) (PNIPAAm) is one of the most attractive thermo-sensitive polymers with a low LCST of approximately 32°C (Wang et al., 2014). For example, Luckanagul reported the synthesis of chitosan-based nanogels with modification by thermo-sensitive PNIPAM and CUR was successfully loaded through a simple sonication method in aqueous media, showing temperature-responsive drug release behaviors (Luckanagul et al., 2018b). In order to study the influence of the degree of crosslinking and the existence of holes in the nanogels on the drug loading and release characteristics, Hajebi synthesized temperature-responsive hybrid core-shell nanogels using NIPAM and vinyl-modified silica nanoparticles *via* precipitation polymerization, and hollow PNIPAM nanogels were obtained by hydrolysis of silicic acid (Hajebi et al., 2020). All results demonstrated that the hollow nanogels had higher DOX loading content and higher toxicity, whereas hybrid nanogels showed faster drug release. Both nanogels presented thermo-sensitive drug release behaviors. The above results show that PNIPAM NG-DDS is promising for cancer treatment due to heat-sensitive, non-toxic, and biocompatible. However, their clinical applications are restricted due to hard degradation of PNIPAM in the body.

Other molecules like acrylamide (AAm) and N-vinylcaprolactam (NVCL) are similar with NIPAM, which exhibit temperature sensitive behaviors. The NVCL-based nanogels through self-assembly with poly (N-vinylpyrrolidone) were successfully prepared, loaded with the non-steroidal anti-inflammatory drug diclofenac sodium (Zavgorodnya et al., 2017). The cumulative transporting amount of drug at 32°C was 12 times than that at 22°C, showing excellent temperature-controlled drug release. Theune developed thermo-sensitive polypyrrole nanogels with spherical shape (200 nm of hydrodynamic size) using semi-interpenetrating *in-situ* polymerization and the obtained nanogels maintained good temperature responsive behaviors in the near-infrared region (Theune et al., 2019). When the MTX was loaded, temperature did not have much effect on the release with the cumulative release amount of 10–15%. However, the release increased significantly with near-infrared radiation, mainly due to that the local heating of the polypyrrole chains weakened the interactions between the drugs and nanogels, causing collapse of nanogels to promote the drug release. Compared with PNIPAM-based nanogels, polypyrrole-based nanogels can be accumulated in multiple intravenous injections without structural collapse at higher temperatures (such as 37°C). Besides, polymers with thermo-sensitivity, such as polyethylene glycol, polyethylene oxide-polypropylene oxide copolymers, poly (ε-caprolactone), and poly (propylene glycol) are also expected to be applied to design temperature-dependent nanogels for drug delivery (Yu et al., 2021).

### Redox-Sensitive Nanogels

**FIGURE 7:**
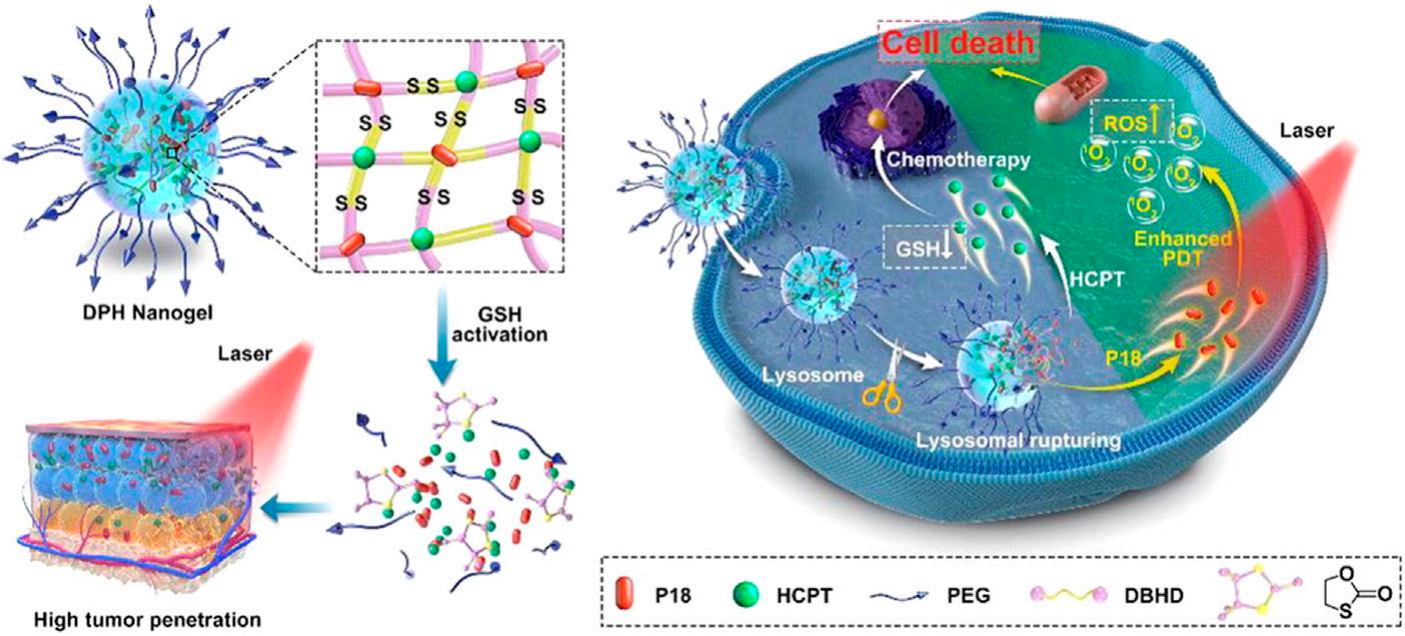
Schematic illustration of redox-responsive prodrug nanogels for combining photodynamic therapy and chemotherapy (Ma et al., 2021). Copyright (2021) Elsevier.

Due to the high proliferation characteristics of tumor cells, high levels of reactive oxygen species (ROS) are overexpressed in tumor tissues and cells, resulting into high levels of reduced glutathione (GSH) in order to maintain redox homeostasis (Wu, 2006). According to research data, the concentration of GSH in tumor cells reaches 2–10 mM, while the concentration in normal tissues is merely around 2–20 μM (Yuan et al., 2018; Kumar et al., 2019). Based on this, Lu developed a two-in-one cross-linking strategy to prepare GSH-responsive prodrug nanogels by coupling DOX and CPT using disulfide compound as cross-linker agent (Lu et al., 2021). Under the high concentration of GSH in tumor, both DOX and CPT released about 75% within 48 h in physiological pH 7.4. Moreover, compared with the single drug, the toxicity of the prodrug nanogels showed significant superiority, indicating the high-performance drug synergetic capacity of this strategy. Similarly, as illustrated in Figure 7, a redox-responsive cross-linker was introduced to synthetize prodrug nanogels based CPT and purpurin 18 (P18) with suitable size (∼67 nm), high drug loading content, controlled drug release, and deep tumor penetration (Ma et al., 2021). According to the release profile, nearly 90% of CPT released in 10 mM GSH, whereas the cumulative release amount was merely 10% without GSH in the pH 7.4 PBS solution. Simultaneously, the released P18 could be activated by NIR of 700–900 nm as a fluorescence imaging agent, demonstrating that nanogels achieved combined photodynamic and chemotherapy as potential drug carrier and diagnostic agent.

Besides disulfide bonds, Se-Se bonds also show stimulus-responsive to redox conditions due to their lower bond energy. More importantly, selenium is an essential trace element for the human body and its anti-cancer activity has been confirmed by anti-cancer mechanisms and clinical experimental studies (Lanfear et al., 1994; Zhu et al., 1996; Moses et al., 2019). For instance, Li and his coworkers chose sodium diselenide as a crosslinking agent to synthesize nanogels with polyphosphate core and hydrophilic PEG shell (Li C. et al., 2015). The nanogels behaved strong inhibitory effects on the proliferation of the tumor cells, which might be attributed to the cleavage of diselenide bonds in the presence of the over-expressed ROS and GSH in the cancer cell. These nanogels were little toxicity to normal cells, and seemed to be promising and highly effective self-release anticancer drug carriers. In addition, the nanogels containing diselenide bonds could also be loaded with other anticancer drugs to achieve combined therapy. For example, the diselenide cross-linked PEG-based nanogels were simply synthesized through physical self-assembly with 32.7% DOX loading content (Hailemeskel et al., 2018). Experiments showed that the initial release was relatively fast in the first few hours with the presence of GSH and H_2_O_2_, reaching 52.5 and 54% at 24 and 48 h, respectively. In the absence of GSH and H_2_O_2_, the 72 h release amount was relatively low at 35.6%. Another important feature of Se is that hydrophobic selenide group can be oxidized to hydrophilic selenoxide/selenone groups with the presence of oxidant. The selenium-containing polyphosphoester nanogels as stable and efficient drug carriers were developed by ring-opening polymerization (Zhang et al., 2018). Under the condition of overproduced ROS in cancer cells (0.05–0.1 mM), the average hydrodynamic diameter of nanogels increased from 164 to 400 nm due to the oxidation of selenide group, which accelerated the efficient intracellular drug release owing to the rapid swelling of nanogels.

### External-Stimulus Responsive Nanogels

The above three stimuli belong to the endogenous stimuli of the tumor microenvironment. In addition, some external stimuli, such as light and magnetic field, are also often used in nanogel drug delivery systems. The UV-light responsive crosslinker 5-(acryloyloxy)-2-nitrobenzyl acrylate was introduced into the methoxy polyethylene glycol methacrylate-based nanogels with higher drug loading and encapsulation efficiency, showing good photo-responsive release capability at the 365 nm ultraviolet light (Xin et al., 2020). Zan developed a near-infrared light-triggered nanogel drug release system based on the advantages of high tissue penetration and low damage. The nanogels loaded with indocyanine green and DOX exhibited superior photo-thermal performance and controlled drug release under NIR laser irradiation (Zan et al., 2015). Besides, the magnetic field-responsive hybrid nanogels have been widely developed recently, which can be attributed to the generation of external heat to kill tumor cells when exposed to a magnetic field. The magnetic/NIR-thermally responsive core-shell hybrid nanogels loaded curcumin were prepared, using bifunctional nanoparticles composed of carbon dot and superparamagnetic nanocrystals cluster as the core and the poly (NIPAM-AAm) as the shell. Both alternating magnetic field and the NIR light irradiation produced local heat, resulting in the shrinkage of poly (NIPAM-AAm) shell and the accelerated release of curcumin. Therefore, the multifunctional hybrid nanogel can be considered as an external stimulus-responsive drug carrier.

### Dual/Multi-Stimuli Responsive Nanogels

At present, the development trend of nanogels as drug carriers is more and more intelligent. Compared with single stimulus-responsive nanogels, the sensitivity and specificity for dual and multiple responsive nanogels to target tumors might be improved (Hajebi et al., 2019a). The physical or chemical properties of these nanogels can be adjusted in response to a stimuli combination of temperature, pH, redox, light, and enzyme to release their cargoes. In this section, we will discuss dual-stimuli and tri-stimuli responsive nanogels and their release behaviors.

**FIGURE 8:**
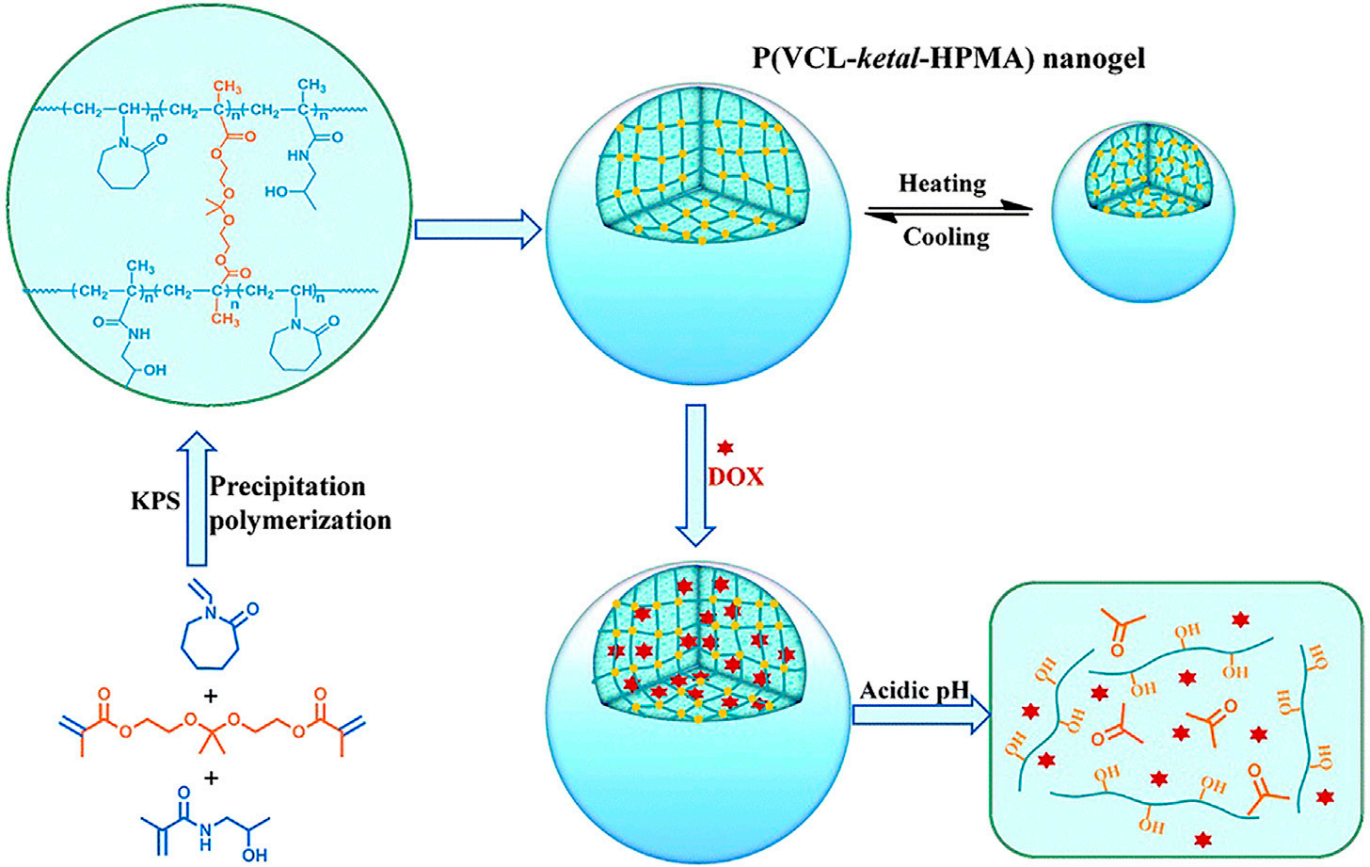
Illustration of the preparation, stimuli-responsive behavior and acid-triggered drug release of the P(VCL-*ketal*-HPMA) nanogel (Wang et al., 2015). Copyright (2015) Royal Society of Chemistry.

Among various dual-stimuli sensitive nanocarriers, the combination of temperature and pH is currently the most widely used. In order to reduce the release of cisplatin in pH-responsive nanogels, NIPAM was introduced into nanogels as the thermo-sensitive unit (Peng et al., 2013). The release of cisplatin in nanogels containing NIPAM was facilitated by H^+^ attack and decreased as the temperature increasing. The results showed that the pH/temperature dual-responsive nanogels are effective intracellular delivery systems for cisplatin drugs. As mentioned above, ketal and PNVCL are common acid and temperature-sensitive compounds. The pH/thermo responsive nanogels loaded with DOX were designed through precipitation polymerization using N-(2-hydroxypropyl) methacrylamide and NVCL as copolymer monomers and the ketal as a cross-linking agent as seen in Figure 8. The release of DOX could be accelerated by lowering the pH and increasing the temperature. Compared with nanogels without ketal agent, the nanogels containing ketal showed obvious acid-dependent release behavior, with a release amount of 13% (pH 7.4) and 96% (pH 5.0) at 48 h, and higher cytotoxicity and efficiency to kill HeLa cells (Wang et al., 2015).

**FIGURE 9:**
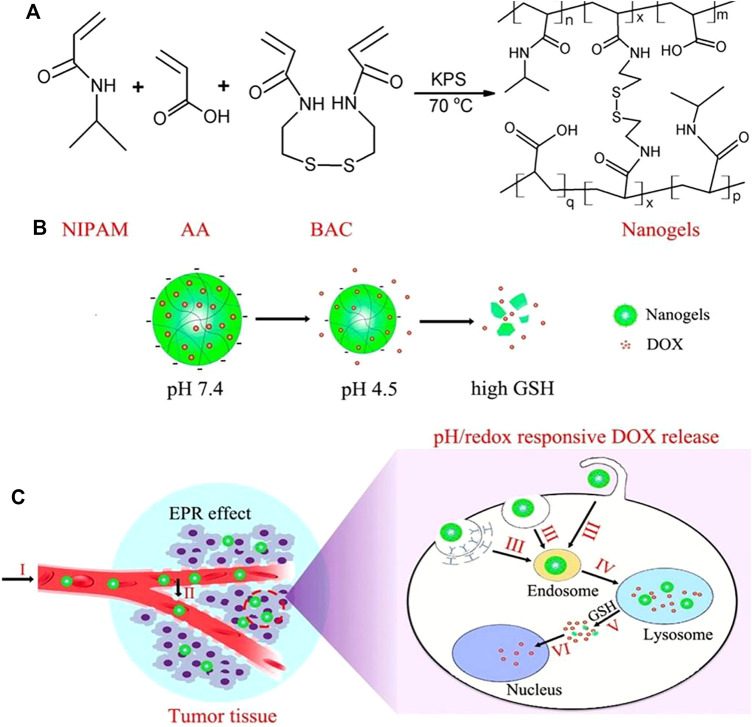
Schematic illustration of P (NIPAM-*ss*-AA) nanogels for anticancer drug delivery. **(A)** Construction of P(NIPAM-*ss*-AA) nanogels; **(B)** Characterization of P(NIPAM-ss-AA) nanogels in response to intracellular microenvironment; **(C)** Site-directed DOX release delivered (Yang et al., 2016). Copyright (2016) American Chemical Society.

Due to the presence of high concentrations of GSH in tumor cells and the acidic environment of the lysosome, pH/GSH dual-sensitive nanogels provide an effective strategy for delivery and intracellular release of anti-cancer drugs (Chen et al., 2014; Li and Liu, 2018). The pH/redox dual-responsive nanogels based on NIPAM and acrylic acid with disulfide bonds as the crosslinking agent were prepared by Yang et al. (2016). As shown in Figure 9, when the DOX-loaded nanogels were internalized into the lysosome of tumor cells, they would shrank to partially release DOX, and then disintegrated under the trigger of high intracellular GSH, causing the complete DOX release. Camptothecin (CPT) belongs to plant anticancer drugs, and has good effects on gastrointestinal, head and neck cancers, but its poor water solubility and serious side effects hinder its clinical application (Ulukan and Swaan, 2002; Tai et al., 2014). To solve these, researchers try to modify the prodrug to improve the performance of CPT. For instance, Qu utilized functionalized CPT, methacrylic acid, and N,N’-methylenebisacrylamide as raw materials, where CPT was chemically coupled to nanogels through disulfide bonds (Qu et al., 2019). The drug release was pH and redox dependent, and it accelerated with the increase of GSH level and the decrease of pH value. At physiological pH 7.4, the cumulative release amount for 48 h was less than 8.6%. However, when the GSH concentration was 2 and 10 mM, the 48 h release amount was 50.9 and 87.7% under the condition of pH 5.0, respectively. It is beneficial to achievie “on-demand” drug release in the microenvironment of tumor cells and tumor tissues for these nanogels.

**FIGURE 10:**
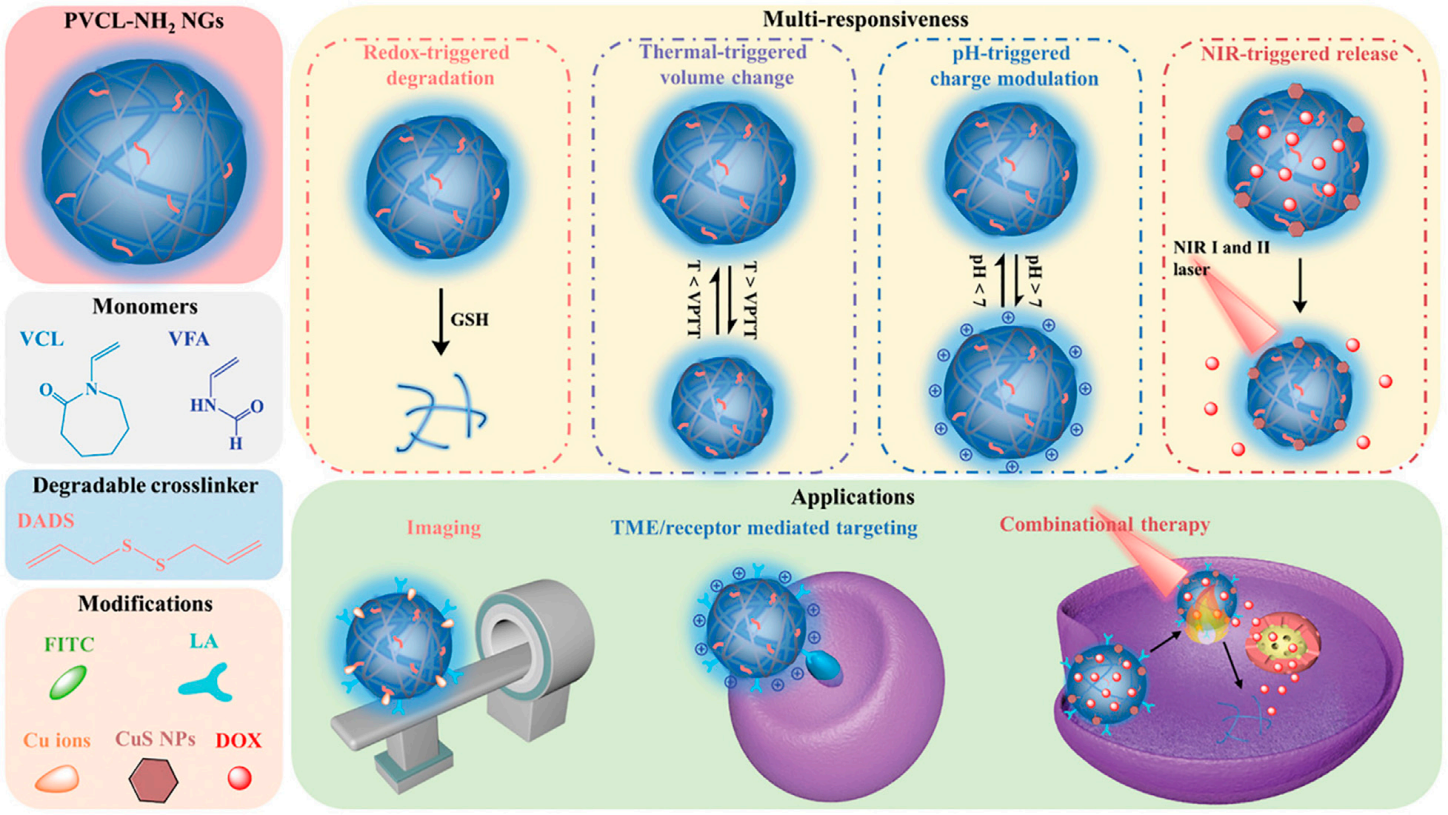
Overview of multi-responsive PVCL-based nanoplatforms (Li X. et al., 2021). Copyright (2021) John Wiley and Sons.

Compared with single-responsiveness and double-responsiveness, multi-responsiveness are hopeful to improve the versatility of carriers to meet more practical needs. According to Lou, a pH/thermo/redox three-stimuli responsive targeted-liver cancer NG-DDS was developed by using functionalized galactose, NVCL, and methacrylic acid as monomers and disulfide bonds compound as a cross-linking agent to encapsulate DOX (Lou et al., 2015). After entering the cancer cells, GSH triggered the cleavage of disulfide bonds and disintegration of nanogels, leading to the release of drugs. PNIPAM was added to the alginate emulsion and cross-linked with cystamine to prepare nanogels with triple-stimuli properties (temperature, pH and redox sensitivity) (Ji et al., 2017). Under the synergistic effects, the release of DOX was more complete than the release of one or two stimuli, which promoted the tumor-targeting and intracellular delivery characteristics of anticancer drug release. Besides the above three stimuli system, light stimulation has also attracted much attention due to the rapid development of photodynamic therapy (PDT) and photothermo therapy (PTT). For example, a new type of disulfide cross-linked polyacrylic acid-methyl spiropyran light/pH/redox responsive drug carrier was designed and synthesized, which tended to be isomerization under ultraviolet light or at low pH, and the addition of the reducing agent dithiothreitol caused collapse of nanogels (Chen et al., 2017). The combined stimulation showed synergistic effect on the DOX cumulative release. Multi-responsive PVCL-based nanogels with core/shell structure were synthesized using precipitation polymerization as exhibited in Figure 10 (Li X. et al., 2021). The nanogels could be employed to modify or/and load with various functional agents to construct multi-stimuli responsive nanoplatforms. DOX-loaded nanogels exhibited pH/NIR/redox triggered drug release, which achieved DOX release of 59.2% under pH 5.5 and NIR laser, and the final DOX cumulative release amount reached 79.2% under pH 5.5 and GSH condition. Remarkably, the combinational therapies are beneficial to the controlled drug release and deeper tissue tumor penetration, achieving enhanced anticancer efficacy compared with single photothermo therapy and chemotherapy.

## Major Challenges and Conclusion

Although there have been lots of research of NG-DDS, there are still problems and challenges in the actual clinical applications. Firstly, the drug loading content of most nanogels is not always high, leading to the low release amount. Therefore, the structures of carriers are required to be further optimized to load more drugs. Secondly, after the carriers entering the blood circulation, the problem of burst release still exists, resulting in significant reduction of drugs that actually target tissues and cells, which requires the deeper exploration of more intelligent stimulus-responsive nanogels to improve the targeting performance and responsiveness. Thirdly, it is currently recognized that the main factor for the enrichment of nanogels in tumor sites is the EPR effect, namely, the high permeability and retention effect due to the rich blood vessels of tumor tissues and the wide vascular wall gap. However, the research on the EPR effect is currently only at the stage of animal experiments and has not been verified in humans. Fourthly, after administration, nanogels are required to overcome lots of biological barriers, such as mucus, skin, tumor microenvironment, blood-brain barrier, etc. Therefore, it is essential to flexibly change the physical and chemical properties of nanogels or modify the surface of nanogels to overcome different biological barriers.

In general, nanogels have shown promising prospects as drug carriers, and provided great potential for intelligent drug delivery. The strategies for designing nanogels as ideal drug carriers should have high drug loading, long circulation time, specific ligands recognized by target cells, and stimulus-sensitive degradation characteristics. There is no doubt that drug carriers are beneficial in tumor treatment, such as reducing drug toxicity, improving efficacy, and enhancing patient tolerance. However, nanogel drug delivery systems can truly achieve clinical applications after solving problems mentioned above.
